# Shaping the next steps of research on frailty: challenges and opportunities

**DOI:** 10.1186/s12877-021-02370-z

**Published:** 2021-07-19

**Authors:** Ivan Aprahamian, Qian-Li Xue

**Affiliations:** 1 Geriatrics Division, Department of Internal Medicine, Jundiai Medical School, Jundiai, Sao Paulo, Brazil; 2 Department of Medicine Division of Geriatric Medicine and Gerontology, School of Medicine, and the Center on Aging and Health, Johns Hopkins University, Baltimore, MD USA

The term “frail elderly” was first introduced by the US Federal Council on Aging under the leadership of Monsignor Charles F. Fahey in the early 1970s [1]. However, until the early 1990s, a frail older adult was stereotyped as someone of old age, having disability/dependency, or multimorbidity; its determination was arbitrary and subjective [2]. In 2001, a seminal paper published by Fried and colleagues defined frailty as a distinct clinical syndrome based on a biological model, and proposed diagnostic criteria characterizing a phenotype of physical frailty (PF) [3]. This phenotypic model of frailty focusing on physical function has since been validated both as a syndromic construct and a robust predictor of relevant adverse health outcomes, independent of age and comorbidity [4]. Around the same time, Rockwood and Mitnitski advanced the deficit accumulation theory to explain and define frailty [5]. Named as the Frailty Index (FI), this instrument can be seen as a biological marker of aging with good mathematical properties [6]. The FI represents a popular tool for risk stratification in various settings; its predictive validity has been established in humans and other species including dogs and mice [7–10]. It is important to note that frailty through the lens of the FI is a multidimensional comorbidity index that is agnostic regarding the correlation, temporal order, and underlying etiology of its composite criteria. As such, the PF and the FI, although sharing the same nomenclature, often yield alarmingly low agreement on frailty classification [11, 12]. While the PF and the FI are the most cited frailty instruments in the research literature, there has been a proliferation of frailty instruments that extend beyond variants of PF and FI to also include subtypes focusing on specific functional domains such as cognitive, psychological, or social frailty [13]. The lack of guidance for proper selection of frailty assessment tool to match goals of care, limited understanding of its biology, and insufficient evidence on interventions have hampered adoption to patients’ health management.

Nonetheless, the steady progress made in the past two decades has helped elevate the importance of frailty in the minds of not only geriatricians but also subspecialists such as cardiologists for whom medical decision-making involving older adults is of common occurrence. In the area of epidemiology, multinational surveys have shown that frailty is a global phenomenon with a prevalence varying between 12 and 24% among community-dwelling older adults worldwide [14]. Evidence of its deleterious impact on health has been consistent, including a two-fold increase in risks of falls and fractures, physical impairment, loss of independence in activities of daily living, hospitalization, and death [15]. Moreover, longitudinal studies revealed exponential trajectory of frailty progression with aging in both sexes and in both frailty models [13], and in the case of physical frailty, a recent study concluded that meeting all five PF criteria signifies “a critical transition toward a point of no return beyond which the process becomes irreversible and death becomes imminent” [16]. Pathophysiologically, recent studies have identified several mechanisms and pathways along the biological hierarchy from physiological (e.g., innate immune system, stress hormones) to the cellular/molecular level (e.g., DNA damage, mitochondria dysfunction) [17, 18]. This strengthens the evidence-base that frailty results from a derangement of the complex homeostatic system compromising one’s ability to withstand intrinsic and extrinsic stressors and ultimately, recover from them. From the perspective of frailty intervention, health behavior interventions (i.e., exercise and nutrition, alone or in combination) and comprehensive geriatric assessment-based and individually tailored interventions have been the primary focus with mixed results [19]. The fact that the existing clinical practical guidelines on management of frailty are largely based on expert opinions in the absence of a high-degree certainty of evidence highlights the urgent need for high-quality randomized controlled trials (RCT) [20].

With the growing interest and urgency for translating frailty research into clinical practice, BMC Geriatrics’ call for papers on frailty represents the latest effort to channel the collective energy of frailty researchers into looking forward and addressing the next generation of research questions. Four topics are worth highlighting here. The first has to do with the issue of primary vs. secondary frailty, with the former being caused primarily by a frayed aging process vs. specific diseases in the latter case. In order to claim that frailty (primary frailty especially) is a unique clinical entity, frailty should not be merely a marker of severity of underlying disease-specific pathology. This is particularly important for studying frailty in the context of specific diseases because the nature of their relationship has direct implications on treatment priorities, e.g., disease-focused treatment in the case of secondary frailty vs. co-management of systemic vulnerability and disease-specific care in the case of primary frailty. In the absence of frailty biomarkers, the disentanglement of frailty and disease processes would require longitudinal follow-up starting ideally at the preclinical stage. It is hopeful that the increasing use of electronic health record systems will make the individual-level tracking of health trajectory increasingly feasible.

Second, there remains an urgent need to balance between the continuing quests for development of therapeutics targeting frailty in its own right, and improving the quality of life of frail individuals by modernizing healthcare delivery systems. For example, care transitions (e.g., hospital to home) represent a point of heightened risk in the care of patients and a significant opportunity to mitigate these safety risks. Given that frailty patients are at high risk for hospitalization and hospital acquired complications such as infection, delirium and falls [21–24], incorporating frailty assessment in the existing care transition models (e.g., Care Transitions Intervention® and Integrated Care Transitions Approach) may help identify the most vulnerable whom may benefit the most from effective care transitions. This is what precision medicine is all about. The value of such intervention for stakeholders of multiple sides will require RCTs that meet high standards of trial design, implementation and measurement, as well as being pragmatic and financially sustainable.

Third, the increasing use of omics-based technologies in frailty research represents a new frontier in a quest to identify novel biological molecules and mechanisms responsible for the generalized physiological dysregulation associated with frailty [25]. However, it is important to recognize that the large number of parameters involved in omics studies raise significant challenges for data analysis, particularly when the number of study subjects is limited. To minimize false positive findings, such analysis needs to take into account reliability of measurement, intra- vs. inter-subject biological variability, the number of parameters being measured relative to sample size, and the prior probability of a hypothesis being true [26]. The last is particularly important, and often is unknown when the primary goal is discovery rather than hypothesis testing. Further, heterogeneity in frailty assessment and causes of frailty (e.g., secondary vs. primary frailty) impairs our ability to discern signal amidst noise (or different signals depending on the cause of frailty) and conduct cross-study comparisons. Therefore, omics studies built on specific hypothesis may be a more fruitful approach. For example, the study of metabolomics related to the mitochondrial tricarboxylic acid (TCA) cycle may generate new insights on the role of dysregulated energetics as a key driver of physiological dysregulation as seen in frailty [27].

Fourth, overcoming individual and societal changes posed by frailty would require integrated solutions beyond medicine. The use of remote sensor technology for health monitoring not only holds the potential to detect subtle changes in the earliest phases of functional decline but also, through high resolution, high frequency, and high volume data acquisition, permits nuanced discovery of clinically meaningful patterns (e.g., fragmented daily physical activity linked to higher risk of death in older adults [28]). It is therefore entirely plausible that the use of such technologies may yield new measures of pre-frailty, representing a preclinical phase of frailty where interventions may be most effective at reversing frailty biology rather than treating frailty symptoms. Another example is the integration of bioengineering methodologies into frailty research including dynamical systems modeling to better understand dysregulated stress-response as a hallmark of frailty [29], and image-based profiling of cell morphologies via machine-learning as a biomarker of cellular aging [30].

In summary, there is an urgent need for an international research agenda to guide frailty research. We must refocus our lens, from focusing mostly on current knowledge, and instead turn our strength and focus into the gaps of frailty science, integrating multiple disciplines to solve complex questions. Future research should move beyond association studies to instead account for heterogeneity in frailty measurement through better alignment of theory and measurement. Identification and testing of causal mechanisms and pathways can be achieved by capitalizing on recent important advances in bioinformatics, engineering, statistics, and technology. Only by utilizing these integrative approaches will frailty research achieve its true value in clinical practice, and translation of research evidence to inform individualized treatment for frail older adults become an achievable goal.
